# Correlation between depression scores and serum NF‐ĸB/NLRP3 axis, biotinidase, and HMGB1 after treatment with isotretinoin in patients with acne vulgaris

**DOI:** 10.1111/jocd.16433

**Published:** 2024-06-24

**Authors:** Ammena Y. Binsaleh, Lahin Saad Ali, Mostafa M. Bahaa, Thanaa A. Elmasry, Walaa A. Negm, Amir O. Hamouda, Muhammed M. Salahuddin, Eman Wahsh, Marwa Ahmed El‐samongy, Nawal Alsubaie, Eman El‐Khateeb, Mohamed Yasser, Nashwa Eltantawy

**Affiliations:** ^1^ Department of Pharmacy Practice, College of Pharmacy Princess Nourah bint Abdulrahman University, P.O. Box 84428 Riyadh 11671 Saudi Arabia; ^2^ Department of Basic Medical Science, Faculty of Dentistry Al‐Ahliyya Amman University Amman Jordan; ^3^ Physiology Department, Faculty of Medicine Mansoura University Mansoura Egypt; ^4^ Pharmacy Practice Department, Faculty of Pharmacy Horus University New Damietta Egypt; ^5^ Pharmacology and Toxicology Department, Faculty of Pharmacy Tanta University Tanta Al‐Gharbia Egypt; ^6^ Pharmacognosy Department, Faculty of Pharmacy Tanta University Tanta Al‐Gharbia Egypt; ^7^ Department of Biochemistry and Pharmacology, Faculty of Pharmacy Horus University New Damietta Egypt; ^8^ Pharmacology and Toxicology Department, Faculty of Pharmacy Sinai University Arish Egypt; ^9^ Dermatology Department, Faculty of Medicine Tanta University Tanta Al‐Gharbia Egypt; ^10^ Clinical Pharmacy Department, Faculty of Pharmacy Tanta University Tanta Al‐Gharbia Egypt; ^11^ Department of Pharmaceutics, Faculty of Pharmacy Port Said University Port Said Egypt; ^12^ Department of Pharmaceutics and Industrial Pharmacy, Faculty of Pharmacy Horus University New Damietta Egypt; ^13^ Pharmacy Practice Department, Faculty of Pharmacy Heliopolis University Cairo 11757 Egypt

**Keywords:** acne vulgaris, biotinidase, depression, HMGB1, NLRP3

## Abstract

**Background:**

Acne vulgaris (AV) is an inflammatory skin disorder leading to scars and discomfort, its intensity has major psychological consequences such as depression.

**Aim:**

To investigate the effect of isotretinoin (ISO) on NF‐κB/NLRP3, biotinidase, and HMGB and correlation with depression.

**Patients and Methods:**

This was a case–control study that involved two groups. Group 1 is 20 healthy control, and group 2 is 20 patients diagnosed with AV according to Global Acne Grading System (GAGS) and received 20 mg ISO for 2 months. Before and after therapy, the Hamilton Depression Rating Scale (HDRS) was applied to assess each participant's level of depression. Nuclear factor kappa B (NF‐ĸB), biotinidase, high mobility group box protein (HMGB1), nucleotide‐binding domain, leucine‐rich–containing family, and pyrin domain–containing‐3 (NLRP–3) were measured in serum samples.

**Results:**

There was no significant difference in all measured markers of healthy group before and after 2 months. Regarding group 2, there was a statistically significant decrease in all measured markers after 2 months of treatment and significant correlations between GAGS, NF‐ĸB, HMGB1, NLRP3, biotinidase, and depression score.

**Conclusion:**

Increased GAGS, HMGB1, NLRP3, and biotinidase were associated with depression severity in AV patients and ISO treatment significantly reduced these parameters and reduced depressive symptoms.

## 1 INTRODUCTION

One of the most prominent skin disorders impacting teenagers worldwide is acne vulgaris (AV), a multifactorial, immune‐mediated, chronic inflammatory illness that affects the hair sebaceous gland units.^1^ The only medication that can permanently cure acne and targets all its primary triggers is isotretinoin (ISO), a potent derivative of vitamin A.^2^ By decreasing the size and production of sebaceous glands, it inhibits the proliferation and the inflammatory activity of keratinocytes in acne and restores the regular maturation of follicular keratinocytes.^3^ Nonetheless, there are two main side effects associated with ISO: teratogenicity and psychiatric issues.^4^ Although ISO's genotoxicity, teratogenicity, and carcinogenicity data were reported to be extremely limited and contradictory,^5^ many patients had been unwilling to utilize ISO because to the reports of its adverse effects.^1^

Studies have revealed that individuals with AV exhibit inflammatory reactions with considerably higher expression levels of CD^4+^ T lymphocytes, macrophages, and interleukin‐1α (IL‐1α) in their normal hair follicles.^6^ There is growing evidence that the pathophysiology of AV involves the nucleotide oligomerization domain (NOD)‐like receptor family pyrin containing 3 (NLRP3) inflammasome.^7^ This inflammasome is known to be triggered by environmental stimuli, endogenous danger signals, and microbial infection. For example, an infection with *Cutibacterium acnes* and *Propionibacterium acnes* can cause a number of inflammatory responses through the generation of inflammatory mediators such NLRP3 and IL‐1β or via NF‐κB/NLRP3 pathway, respectively.^7^, ^8^

Inflammation can also trigger the release of the intracellular highly conserved protein known as high‐mobility group box 1 (HMGB1) that is present in most cells in the body. HMGB1 in turns attaches to Toll‐like receptors 2 or 4 or the receptor for advanced glycation end products (RAGE) to exert pro‐inflammatory reactions.^9^ Acne etiology has been linked to HMGB1, and there is a correlation between the disease's severity and HMGB1 level.^10^

Biotinidase, an enzyme essential to intestinal biotin absorption, can cause a biotin deficit.^11^ There is a suggestion that reduced biotinidase activity is caused by ISO isomers and metabolites acting in the liver.^12^ The negative effect of isotretinoin metabolites on the liver is assumed to lower biotinidase activity. Skin fragility and alopecia, two adverse reactions linked to ISO medication, may be caused by a partial biotinidase deficiency.^13^

Compared to the prevalence observed in the general population, the incidence of depressive disorders among dermatological patients can reach up to 25%–40%.^14^ Up to 95% of individuals experience acne scarring, which can still occur even after receiving appropriate and timely therapy. Acne scarring is a very problematic acne‐related condition. It has been established that the patients' psychological health and quality of life are negatively impacted by the obvious depressed scars. Scarring from acne can increase one's risk of despair, anxiety, and even suicide.^15^ Since there is growing evidence between mental health issues to acne, the psychological effects of acne have been thoroughly investigated.^16^

Based on this evidence, the current research aimed to assess the effect of ISO on serum NLRP3, HMGB1, Global Acne Grading System (GAGS), and biotinidase and correlate them with depression.

## 2 PATIENTS AND METHODS

This was a prospective case–control study that was conducted during the period from August 2023 to February 2023. The study included 20 outpatient clinic and 20 healthy control who met the inclusion criteria. The design and methodology of the study adhered to the 1964 Helsinki Declaration and its later revisions. It was explained to the patients that they were free to discontinue the trial at any moment. Prior to participating in this trial, patients gave their signed, informed consent.

### 2.1 Inclusion criteria for diseased patients

Acne patients included in the study were fulfilling the following criteria:

- Adult patients, male or female (18–35 years).
- Body mass index (BMI) ranging from 18.5 to 23 kg/m^2^.
- Scoring more than 18 points for global acne.

### 2.2 Inclusion criteria for healthy control

Healthy subjects in the study were enrolled after fulfilling the following criteria:

- Adult patients (18–35 years old), male or female.
- BMI from 18.5 to 23 kg/m^2^.
- Absence of acne symptoms.
- Exclusively in women, regular monthly cycle for the previous 3 months.

### 2.3 Exclusion criteria

Subjects were excluded from the study if any of the following criteria were present:

- Breastfeeding and/or pregnant women.
- Excessive alcohol consumption.
- Medical diseases or conditions (depression, chronic liver or kidney disease, hyperlipedemia and thyroid conditions).
- Intolerance to ISO.
- Use of antidepressants, oral antibiotics, herbal remedies, and oral vitamin A derivatives.
- Patients on medications for acne that include corticosteroids (either oral, injectable, or topical).
- Patients on anabolic steroids, phenytoin, lithium, isoniazid, cyclosporin, iodides, bromides, and azathioprine within the previous four weeks that can have an impact on the test results.
- Taking part in other clinical studies.

### 2.4 Study intervention

This was a prospective case–control study that investigated at the safety and efficacy of ISO in the management of AV patients. This research was registered as NCT06242288 on WWW.ClinicalTrials.gov. The study included 20 patients with AV and 20 healthy control who were screened according to CONSORT diagram (Figure 1).

**FIGURE 1:**
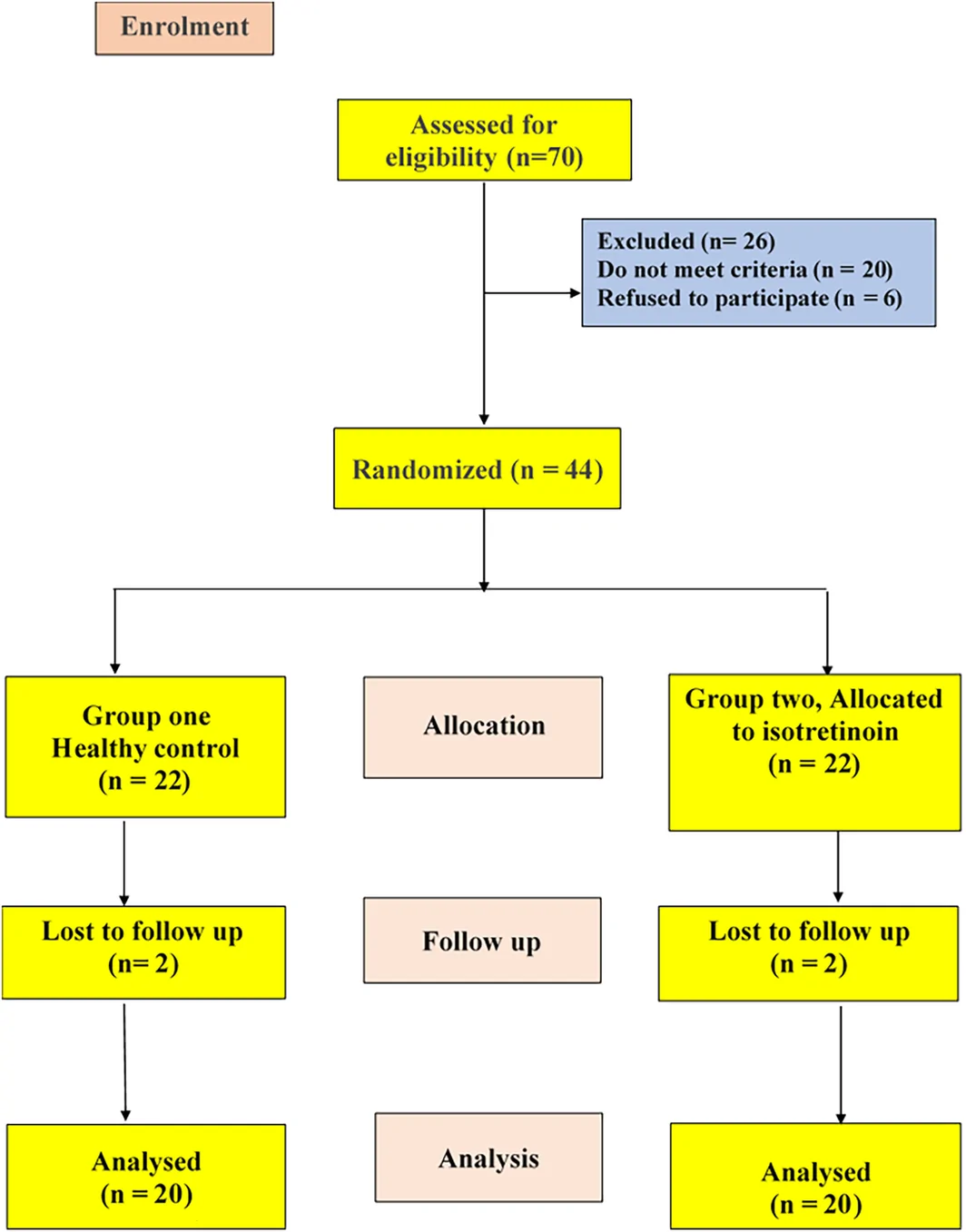
CONSORT diagram showing participation of subjects during the study.

Group 1: 20 healthy control subjects. They did not receive any medication over the duration of the study (2 months).

Group 2: 20 patients were given 20 mg ISO once daily for 2 months (Netlook® 10 mg, Andalous Pharma, Egypt).

### 2.5 Evlauation of AV and depression scores

Patients were evaluated for acne by GAGS.^17^ This particular system was selected due to its ability to offer thorough and precise information regarding the severity of acne, as well as aiding in the identification of different forms of acne and the location of lesions. The following local outcomes make up the global score: There are four categories of acne: mild,^1^, ^2^, ^3^, ^4^, ^5^, ^6^, ^7^, ^8^, ^9^, ^10^, ^11^, ^12^, ^13^, ^14^, ^15^, ^16^, ^17^, ^18^ moderate,^19^, ^20^, ^21^, ^22^, ^23^, ^24^, ^25^, ^26^, ^27^, ^28^, ^29^, ^30^ severe,^31^, ^32^, ^33^, ^34^, ^35^, ^36^, ^37^, ^38^ and very severe (over 39).

Also, all participants were evaluated for depression by Hamilton Depression Rating Scale (HDRS) at the baseline and after 2 months.^18^ The most popular depression assessment measure used by clinicians is the Hamilton Depression Rating measure (HDRS, commonly called the Ham‐D). The original edition of the HDRS has 17 items (HDRS17) related to depressive symptoms experienced in the previous week. The HAMD has four severity ranges: mild depression,^8^, ^9^, ^10^, ^11^, ^12^, ^13^, ^14^, ^15^, ^16^ moderate depression,^17^, ^18^, ^19^, ^20^, ^21^, ^22^, ^23^ severe depression (≥24), and no depression (0–7).

### 2.6 Biochemical analysis

Every patient or participant provided serum samples at the baseline (day 1) and after 2 months of the intervention. Enzyme‐Linked Immunosorbent Assay (ELISA) method and kits were used in accordance with the manufacturer's instructions (Sunredio, Shanghai, China) for measuring serum NLRP3 (kit catalogue no. 201‐12‐5748), serum HMGB (kit catalogue no. 201‐12‐1636), NF‐kB (kit catalogue no. 201‐12‐0691) in these samples. Biotinidase was also measured using ELISA kit with catalogue no: SL2349Hu, supplied by SunLong Biotech Co., LTD.

The laboratory tests for low‐density lipoprotein (LDL; mg/dL), triglycerides (TG; mg/dL), high‐density lipoprotein (HDL; mg/dL), total cholesterol (TC; mg/dL), aspartate and alanine transaminases (ALT and AST, respectively) were also measured using enzymatic colorimetric techniques. All patients were followed up weekly to ensure compliance with the treatment regimen and assess any adverse effects up to 2 months from starting the intervention.

### 2.7 Statistical analysis

Version 9 of GraphPad Prism was used to do the statistical analysis (GraphPadsoftware, Inc., San Diego, CA). The Shapiro–Wilk test was conducted to normalize the data. Paired Student's *t*‐test was used to find significant differences within groups before and after 2 months of the intervention. Any significant changes between the groups before and after the intervention were assessed using an unpaired Student's *t*‐test. All the data were displayed as the mean ± SD, numbers, and the interquartile range (IQR). Both the Fisher exact test and the chi‐square test were utilized in statistical analyses of categorical data as appropriate. The Pearson correlation test was used to analyze the correlation between the measured parameters. Two‐tailed *p* < 0.05 was regarded as statistically significant.

## 3 RESULTS

### 3.1 Clinical and demographic data in the two study groups

Table 1 displays baseline statistics of participants. There were no significant differences in demographic data between the studied groups; age (*p* = 0.963), sex (*p* = 0.438), and BMI (*p* = 0.527).

**TABLE 1:** Comparison of the demographic and anthropometric data between the two study groups at baseline.

Parameter	Group 1	Group 2	*p*‐value
Age (years)	27.05 ± 3.692	27.00 ± 3.179	0.963
Sex (male/female)	11/9	14/16	0.438
BMI (kg/m^2^)	22.70 ± 1.376	22.43 ± 1.240	0.527

*Note*: Data are expressed as mean ± SD, and numbers. Significance at (*p* < 0.05). Group 1, 20 healthy control. Group 2, 20 patients received isotretinoin 20 mg for 2 months.

Table 2 showed that 4 patients have family history of AV, and 16 did not have. Concerning previous treatment with ISO: 18 patients did not receive previous treatment, while only 2 patients received it.

**TABLE 2:** Clinical and descriptive data of Group 2 patients.

Parameter	Category	Group 2 (*n* = 20)
Previous treatment with isotretinoin	No	18 (90.9%)
	Yes	2 (9.1%)
Duration	Median (IQR)	1.55 (1–2)
Family history	Yes	4 (20%)
	No	16 (80%)

*Note*: Data are expressed as numbers, percentage, median and interquartile range (IQR). Group 2, 20 patients received isotretinoin 20 mg for 2 months.

### 3.2 Analysis of serum biomarkers and Hamilton Depression Rating Scale in the two study groups

Using paired *t*‐test, Table 3 shows that the healthy control group did not have any statistically significant differences in all measured markers before and after 2 months of starting the study as follows: NF‐κB (*p* = 0.159), HMGB1 (*p* = 0.652), NLRP‐3 (*p* = 0.177), biotinidase (*p* = 0.407), and HDRS (*p* = 0.427).

**TABLE 3:** Analysis of serum biomarkers and Hamilton Depression Rating Scale in the two study groups.

Character	Group 1			Group 2			^b^ *p*‐values	
	Baseline	After 2 months	^a^ *p*‐values	Baseline	After 2 months	^a^ *p*‐values	Baseline	After 2 months
NF‐κB (pg/mL)	3.923 ± 1.020	4.385 ± 0.615	0.159	16.98 ± 1.827	4.495 ± 6.211	0.0001	0.0001	0.576
HMGB1 (pg/mL)	124.2 ± 9.901	122.8 ± 7.820	0.652	151.3 ± 10.04	122.0 ± 11.12	0.0001	0.0001	0.806
NLRP‐3 (pg/mL)	145.3 ± 10.21	143.3 ± 10.28	0.177	162.6 ± 12.19	149.3 ± 8.602	0.0001	0.0001	0.149
Biotinidase (ng/mL)	1620 ± 32.77	1639 ± 89.43	0.407	1641 ± 76.76	682.0 ± 42.85	0.0001	0.281	0.0001
HDRS	3.850 ± 2.15	3.400 ± 2.280	0.427	15.70 ± 2.250	4.450 ± 2.038	0.0001	0.0001	0.133

*Note*: Data are expressed as mean ± SD, significance at (*p* < 0.05). Group 1, 20 healthy control. Group 2, 20 patients received isotretinoin 20 mg for 2 months.

Abbreviations: HDRS, Hamilton Depression Rating Scale; HMGB1, high mobility group box 1; NF‐κB, nuclear factor kappa B; NLRP‐3, nucleotide‐binding domain, leucine‐rich–containing family, pyrin domain–containing‐3.

^a^ Within‐group comparison.

^b^ Between‐group comparison.

Regarding AV group, paired *t*‐test showed that there was a statistically significant difference in all measure markers before and after 2 months of the ISO treatment intervention as follows: NF‐κB (*p* = 0.0001), HMGB1 (*p* = 0.0001), NLRP‐3 (*p* = 0.0001), biotinidase (*p* = 0.0001), and HDRS (*p* = 0.0001).

Unpaired *t*‐test revealed that there was a significant difference in all measured markers between baseline values of the two groups (*p* = 0.0001) except for no significant difference in baseline values of biotinidase (*p* = 0.281). However, 2 months of the intervention, unpaired *t*‐test displayed no significant difference in all measured markers (*p* > 0.05) except for biotinidase (*p* = 0.0001).

### 3.3 Analysis of lipide profile and liver enzymes in both groups

Before treatment, Table 4 showed that there were no significant differences between baseline values of liver enzymes and lipid profile in both groups using unpaired *t*‐test (*p* > 0.05).

**TABLE 4:** Analysis of lipide profile and liver enzymes in both groups.

Character	Group 1			Group 2			^b^ *p* value	
	Baseline	After 2 months	^a^ *p* value	Baseline	After 2 months	^a^ *p* value	Baseline	After 2 months
ALT (IU/L)	54.25 ± 8.663	53.05 ± 8.556	0.372	54.10 ± 8.485	77.70 ± 10.80	0.0001	0.956	0.0001
AST (IU/L)	49.80 ± 12.85	48.00 ± 12.05	0.377	44.55 ± 5.557	73.40 ± 7.95	0.0001	0.096	0.0001
TC (mg/dL)	148.1 ± 14.77	149.4 ± 16.35	0.717	150.7 ± 18.38	237.6 ± 17.99	0.0001	0.624	0.0001
TG (mg/dL)	125.8 ± 15.1	124.7 ± 14.32	0.079	127.4 ± 12.75	162.8 ± 15.11	0.0001	0.716	0.0001
LDL (mg/dL)	82.55 ± 12.43	85.40 ± 10.70	0.109	81.60 ± 12.19	174.0 ± 13.55	0.0001	0.808	0.0001
HDL (mg/dL)	74.95 ± 7.323	79.20 ± 8.063	0.129	71.30 ± 6.071	59.20 ± 5.167	0.0001	0.094	0.0001

*Note*: Data are expressed as mean ± SD, significance at (*p* < 0.05). Group 1, 20 healthy control. Group 2, 20 patients received isotretinoin 20 mg for 2 months.

Abbreviations: ALT, Alanine transaminase; AST, Aspartate transaminase; HDL, High‐density lipoprotein; LDL, Low‐density lipoprotein cholesterol; TC, Total cholesterol; TG, Triglycerides.

^a^ Within‐group comparison.

^b^ Between‐group comparison.

Regarding healthy control group, no significant differences were observed in all measured markers (ALT, AST, TC, TG, HDL, LDL) by paired *t*‐test (*p* > 0.05).

Regarding AV group, paired *t*‐test showed that there were significant differences between before and after 2 months of treatment in all measured parameters (*p* > 0.0001). After treatment, unpaired *t*‐test showed that patients in the AV group have significantly higher values of liver enzymes and lipid profile when compared to healthy control (*p* > 0.0001).

### 3.4 Analysis of isotretinoin side effects in AV group

After 2 months of treatment, 11 patients developed dry skin, 9 patients developed cracking lips, and 5 patients had nausea.

### 3.5 Effect of isotretinoin on GAGS

After 2 months treatment with ISO, the GAGS was significantly reduced (33.25 ± 6.008 vs. 20.20 ± 5.217, *p* ≤ 0.0001).

### 3.6 Correlation analysis between the measured markers in AV group

After 2 months of treatment with ISO, there was a significant correlation between GAGS and HDRS (*r* = 0.79), GAGS and NF‐ĸB (*r* = 0.8), GAGS and biotinidase (*r* = 0.754), and GAGS and HMGB1 (*r* = 0.638) (Figure 2).

**FIGURE 2:**
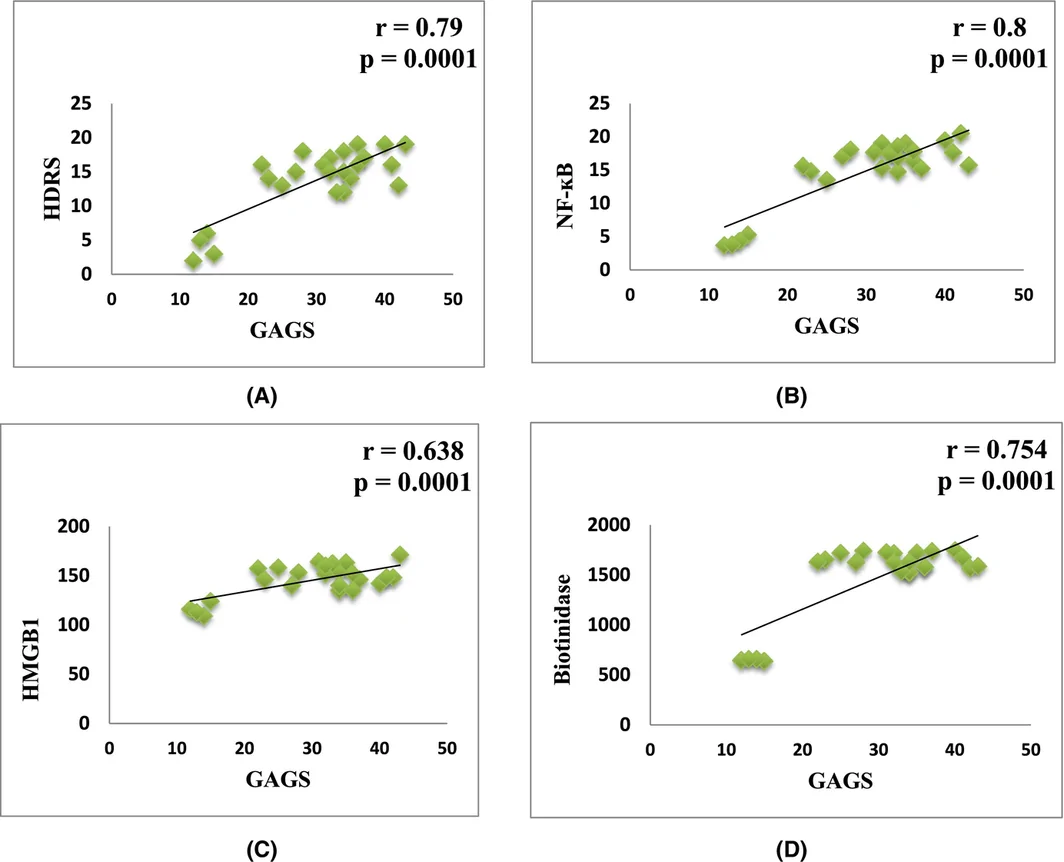
Correlation analysis between (A) GAGS and HDRS, (B) GAGS and NF‐κB, (C) GAGS and HMGB1, and (D) GAGS and biotinidase.

Regarding correlation of depression and AV and the measured markers, there was a significant correlation between HDRS and biotinidase (*r* = 0.936), HDRS and HMGB1 (*r* = 0.739), and HDRS and NF‐ĸB (*r* = 0.926) (Figure 3).

**FIGURE 3:**
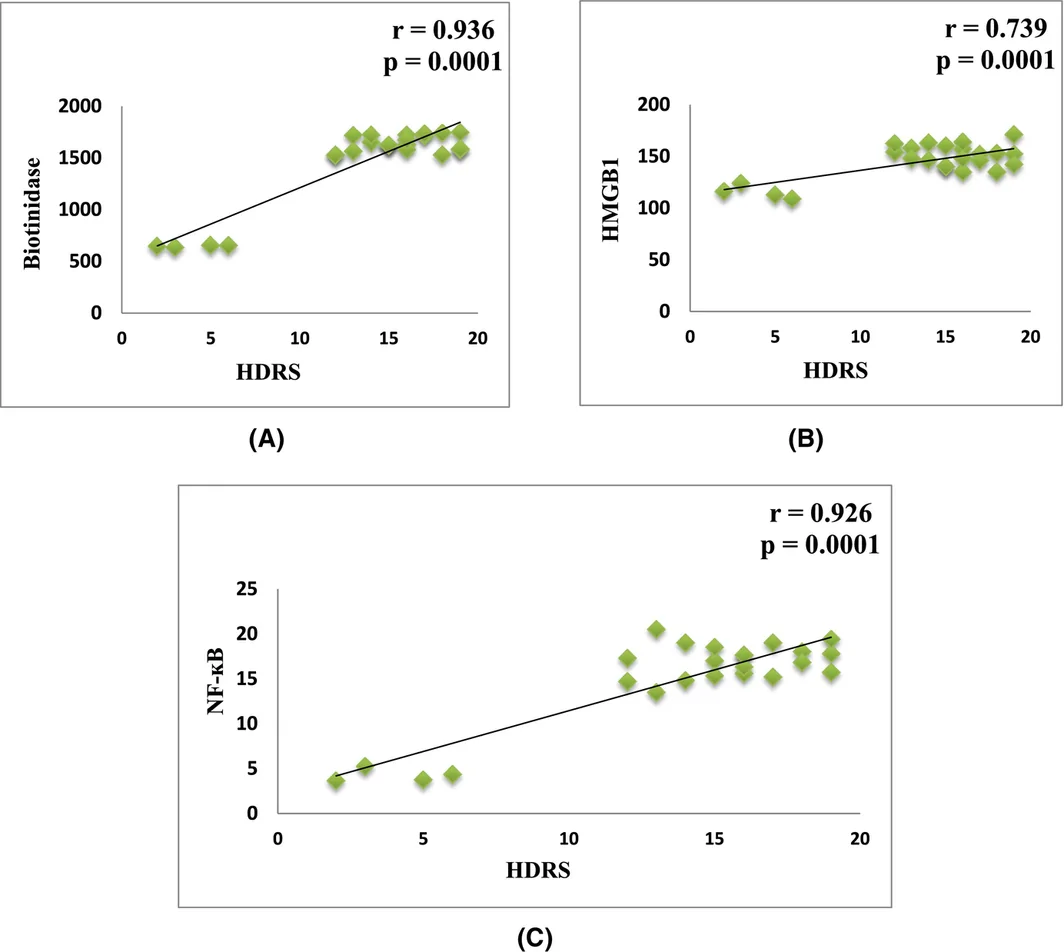
Correlation analysis between (A) HDRS and biotinidase, (B) HADRS and HMGB1, and (C) GAGSHDRS and NF‐κB.

## 4 DISCUSSION

AV is a worldwide chronic inflammatory skin condition that impacts the pilosebaceous follicles.^19^ Acne is predicted to affect 4% of the global population, ranking it as the ninth most frequent skin disorder.^20^

It is well known that AV is an inflammatory condition and induce state of depression and there is a strong correlation between the severity of depression and the degree of acne.^21^ The inflammatory pathways are involved in the pathogenesis of depression.^22^ Evidence exists to support the close correlation between the neurological and immune systems.^22^ The central nervous system (CNS) can be affected directly or indirectly by inflammations, whether they are acute or chronic. It has been found that both individuals with depression and rodent depression models have active NLRP3 inflammasomes.^22^ Stress‐induced depression requires a functional NF‐ĸB/NLRP3 axis, whereas NLRP3 inhibition counteracts these stress‐like effects.^22^ Thus, targeting the NF‐ĸB/NLRP3 axis and the correlation between these markers and depression score could be a therapeutic strategy for treating ISO‐induced depression.

Acne itself has been demonstrated in earlier research to have detrimental impacts on quality of life, which is similar to other chronic conditions.^23^ Our study confirmed the strong correlation between HDRS and GAGS and reported by a previous study.^24^ Additionally, the current study showed that there was a significant elevation in GAGS and HDRS in AV patients and its level were significantly reduced after treatment with ISO. ISO treatment decreased GAGS and scars that may affect face shape and leading to social isolation.^25^ There was a significant correlation between HDRS and HMGB1, NF‐ĸB, and NLRP3. Furthermore, there was a significant correlation between GAGS and NLRP3, HMGB1, and NF‐ĸB. These observations speculate that increasing acne severity is usually associated with increased inflammatory response manifested by these results.^26^ After treatment with ISO, reduction in GAGS, HDRS, NLRP3, HMGB1, and NF‐KB was observed. These results imply that ISO was effective in decreasing sebum production, decreased keratinization, and hence decreased inflammatory pathways associated with the pathophysiological events of AV.^27^

While some studies have linked ISO to adverse effects like depression, a recent well‐controlled large prospective study has not found a connection between ISO and depression.^23^ Our study revealed that the use of ISO decreased HDRS and alleviated depressive symptoms and psychiatric status of patients. This observation may be due to that ISO has anti‐inflammatory activity and reduces the inflammatory pathways. Other studies reported that ISO treatment led to depression and suicidal thoughts,^28^ especially in patients with family history of depression and no improvement in acne score. Other reports showed that acne patients' quality of life was enhanced by ISO medication, but there was some increased depression in the patients.^23^

In our study we revealed that AV patients have rising levels of inflammatory parameters such as NF‐ĸB, NLRP3, and HMGB1 when compared to healthy control. These observations were in agreement with previous studies.^10^, ^29^, ^30^ After treatment with ISO, these biomarkers were significantly reduced. These results match with previous researches.^30^, ^31^ These observations might be explained as follows: ISO has anti‐inflammatory properties, enhances keratinization, lowers sebum production, and decreases the amount of *C. acnes*.^27^ Moreover, ISO blocks the NF‐ĸB transcriptional factor via blocking the Toll‐like receptor signaling pathway.^32^, ^33^ Consequently, NF‐ĸB regulates the expression of genes that produce inflammatory mediators, which in turn produce a variety of cytokines, chemokines, adhesion molecules, and other growth factors.^32^ Furthermore, ISO inhibits inflammation by decreasing the activity of *P*. *acnes*.^34^ *P*. *acnes* causes inflammation in acne by stimulating keratinocytes, monocytes, sebocytes, and inflammatory cells; it also causes these cells to release pro‐inflammatory mediators such IL‐8, IL‐1β, IL‐6, HMGB1, NF‐ĸB, and tumor necrosis factor (TNF)‐α.^34^, ^35^ By suppressing NF‐ĸB, blocking mammalian target of rapamycin (mTOR) and activating adenosine monophosphate‐activated protein kinase (AMPK) ISO has an effect on NLRP3.^36^ ISO lowers NF‐ĸB and toll‐like receptors, which results in the indirect reduction of HMGB1.^37^, ^38^ These observations might explain the inhibitory effect of ISO on serum NF‐ĸB/NLRP3 and HMGB1.

The biotinidase level in the AV group was considerably lower after ISO treatment than it was at baseline. These observations are in line with other researches.^12^, ^39^ Consuming ISO disrupts the balance of the gut flora, leading to malabsorption and the potential for acquired biotin deficiency. Low biotinidase activity may be caused by ISO isomers and metabolites acting in the liver. Partial biotinidase deficiency may be linked to certain dermatological adverse effects associated with ISO, such as alopecia and skin fragility.^12^ Reduced activity of biotinidase, an enzyme essential to intestinal biotin absorption, can also cause a biotin deficit. It was discovered that the liver was most likely the source of biotinidase in serum. Additionally, the lower limit of activity identified in healthy people was observed in the serum biotinidase activities of patients with chronic hepatic disorders.^40^ As demonstrated by our research and other findings, ISO reduced liver function to create biotinidase by increasing hepatic enzyme levels and decreasing liver activity after 2 months of initiating the therapy. Serum biotinidase was normal in both groups at baseline and dropped during ISO medication, which could be explained by this indirect mechanism.

Analysis of side effects revealed that there was a significant elevation in lipid profile and liver enzymes in AV group. Also, ISO treatment resulted in dry skin and dry lips. These results were in line with previous reports.^41^, ^42^, ^43^ Literature research has also revealed that elevated hepatic enzyme levels brought on by retinoid therapy are often mild to moderate and do not result in long‐term liver damage and this side effect is reversible.^43^ Transaminase levels are changed by ISO, possibly as a result of changes in liver inflammation. Two to four weeks after stopping the medication, the symptoms disappeared, demonstrating the safety of retinoids.^44^

Our study had certain limitations, including an inadequate sample size, a short treatment and follow‐up period, and a lack of variation in ISO doses. Furthermore, this study is limited to the eastern population of Egypt and is monocentric.

## 5 CONCLUSION

The study investigated the effect of ISO on NF‐κB/NLRP3, biotinidase, and HMGB, and its correlation with depression in patients with AV. These findings suggest that ISO effectively modulates the inflammatory markers associated with AV, potentially contributing to the improvement of the skin condition. Furthermore, this correlation underscores the complex interplay between inflammatory pathways, skin health, and psychological well‐being in AV patients.

Overall, the results support the use of ISO as a promising therapeutic option for AV, not only for its direct impact on inflammatory markers but also for its potential to alleviate the psychological burden associated with the condition. Further research in this area with a longer follow‐up period could provide additional insights into the mechanisms underlying the efficacy of ISO, its safety, and its broader implications for the management of AV and associated psychological comorbidities.

## AUTHOR CONTRIBUTIONS

Conceptualization: A.Y.S., T.A.E., M.M.B., and N.E.L. Study design: W.A.N., A.O.H., E.E., and M.M.B. Data analysis: M.M.S., E.W.A., A.M.A., and N.E.L. Writing original draft: W.A.N., A.O.H., E.E., and M.M.B. Validation: A.Y.S., T.A.E., M.M.B., M.M.S. and N. A. L. Software: W.A.N., A.O.H., M.M.B., A.M.A., and M.M.B. Data curation: A.Y.S., T.A.E., M.M.B., M.A.E.L and N. A. L. Supervision: N.E.L., M.M.S., E.E. and M.M.B. Writing, review, and editing: A.Y.S., T.A.E., M.M.B., N. A. L, W.A.N., E.E., L.S.A., M.Y. and N.E.L. Revision and proofreading: E.E., M.Y. and L.S.A.

## FUNDING INFORMATION

Nothing to declare.

## CONFLICT OF INTEREST STATEMENT

The authors have no conflicts of interest.

## ETHICS STATEMENT

Institutional Review Board of Tanta University's Faculty of Medicine amended the study protocol and approved it after reviewing it for all ethical and academic issues.

## CONSENT

The informed consent was given by each study participant.

## Data Availability

The data that support the findings of this study are available on request from the corresponding author. The data are not publicly available due to privacy or ethical restrictions.
